# Trophic facilitation in forest restoration: Can *Nothofagus* trees use ectomycorrhizal fungi of the pioneer shrub *Leptospermum*?

**DOI:** 10.1002/ece3.11442

**Published:** 2024-05-26

**Authors:** Merissa Strawsine, Laura G. van Galen, Janice M. Lord, Matthew J. Larcombe

**Affiliations:** ^1^ Department of Botany University of Otago Dunedin New Zealand; ^2^ Department of Environmental Systems Science ETH Zürich Zürich Switzerland; ^3^ Society for the Protection of Underground Networks (SPUN) Dover Delaware USA; ^4^ Present address: Shasta‐Trinity National Forest Redding California USA

**Keywords:** facilitation, home soil advantage, mycorrhizal fungi, Myrtaceae, Nothofagaceae, restoration, symbiosis

## Abstract

The benefits of plant‐to‐plant facilitation in ecological restoration are well recognized, yet the potential for indirect trophic facilitation remains understudied. *Nothofagus* (southern beech; Nothofagaceae) is an iconic southern hemisphere tree genus that is frequently the focus of ecological restoration efforts. One aspect of *Nothofagus* ecology that may limit restoration success is the availability of appropriate ectomycorrhizal fungi. It has been suggested that pioneer dual‐mycorrhizal hosts such as *Leptospermum* species (Myrtaceae) could facilitate *Nothofagus* establishment by providing fungal inoculum, but the capacity for *Nothofagus* to use *Leptospermum* ectomycorrhizal fungi is unknown. To investigate potential indirect facilitation, we conducted a common garden pot trial to determine if *Nothofagus cliffortioides* (mountain beech) can use symbionts from *Leptospermum scoparium* (mānuka) ectomycorrhizal communities. *Nothofagus* and *Leptospermum* seedlings were grown in monoculture and mixed pairs with reciprocal “home” and “away” soil fungal inoculum. ITS2 metabarcoding of eDNA from hyphal ingrowth bags revealed that *Nothofagus* and *Leptospermum* inoculum contained different ectomycorrhizal fungal communities, but that half of the common ectomycorrhizal taxa identified were found in both soil types, suggesting generalist fungi exist. *Nothofagus* was able to form associations with some fungal species originating from *Leptospermum* inoculum, however, probable spore contamination meant that the proportion of root colonization associated with those species was ambiguous. Root ectomycorrhizal colonization rates were positively associated with seedling biomass, and there was some evidence of a home soil inoculum advantage in *Nothofagus*, but these effects were minor. Additionally, we found evidence that home inoculum provides a protective advantage against drought stress for *Leptospermum* seedlings. Our results indicate the potential for using *Leptospermum* to promote *Nothofagus* establishment in restoration plantings and highlight the possible benefits of considering fungal mutualists in ecological restoration projects.

## Graphical abstract

We show the potential to improve restoration success through trophic facilitation. The hard‐to‐establish canopy species *Nothofagus cliffortioides* can utilize the ectomycorrhizal fungi associated with the pioneer shrub *Leptospermum scoparium*. The results demonstrate that preplanting *L. scoparium* could improve restoration of *N. cliffortioides* by improving the fungal symbiont community.

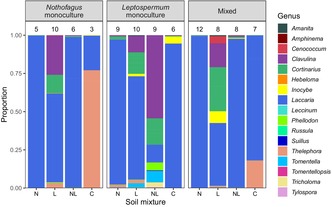

## 1 INTRODUCTION

Ecological restoration is critical for reversing trends in forest degradation and reinstating lost ecosystem services (Gann et al., 2019). The United Nations *Decade of Ecosystem Restoration* aims to restore 350 million hectares of degraded landscapes globally by 2030 (United Nations Environment Programme, 2020). However, despite excellent restoration outcomes in some situations, the on‐ground experience is often highly variable. Many projects fail to produce the desired outcomes, especially when dealing with highly degraded and/or stressful environments (Miller et al., 2017; Svejcar et al., 2017). Improving restoration outcomes will likely require the reestablishment of complex ecological processes such as trophic interactions and food webs (Fraser et al., 2015; Kardol & Wardle, 2010; Miller et al., 2017).

Plant‐to‐plant facilitation has been widely used to improve restoration outcomes (e.g., Gómez‐Aparicio et al., 2004; Tulod & Norton, 2020a). However, this has focused mostly on non‐trophic effects, such as the use of nurse plants, where plant‐to‐plant interactions provide net benefits by ameliorating environmental stressors. Indirect trophic facilitation, where one organism positively influences another by affecting organisms at another trophic level, has received much less attention (Filazzola & Lortie, 2014). For example, a pioneer plant species may alter the heterotrophic microbial community at a restoration site, facilitating the establishment of later‐successional species (Kardol et al., 2013). Research in restored prairie grasslands has shown that changes in soil communities can drive the successional trajectory of the plant communities due to positive plant–arbuscular mycorrhizal feedback loops (Koziol & Bever, 2019; Middleton & Bever, 2012), and plant and fungal community composition is often closely linked along successional gradients in ectomycorrhizal systems (Li et al., 2020; Qiang et al., 2021; Zhao et al., 2023). It, therefore, seems feasible that trophic interactions could improve mycorrhizal conditions to affect plant function and improve restoration outcomes (Bahadur et al., 2019). For example, drought stress is a major limiting factor during restoration establishment (Shackelford et al., 2021), and despite evidence that mycorrhizal inoculation improves drought resistance in agriculture and forestry (Ortega et al., 2004; Tang et al., 2022), research is limited in restoration settings and the results have been mixed (Chaudhary et al., 2020; Zhang et al., 2019). Understanding how trophic interactions could be harnessed to benefit mycorrhizal communities and restoration practice is complicated by the difficulty of studying complex microbial interactions in a restoration setting. Here, we present a case study investigating the potential for indirect trophic facilitation in forest restoration in New Zealand.

**FIGURE 1:**
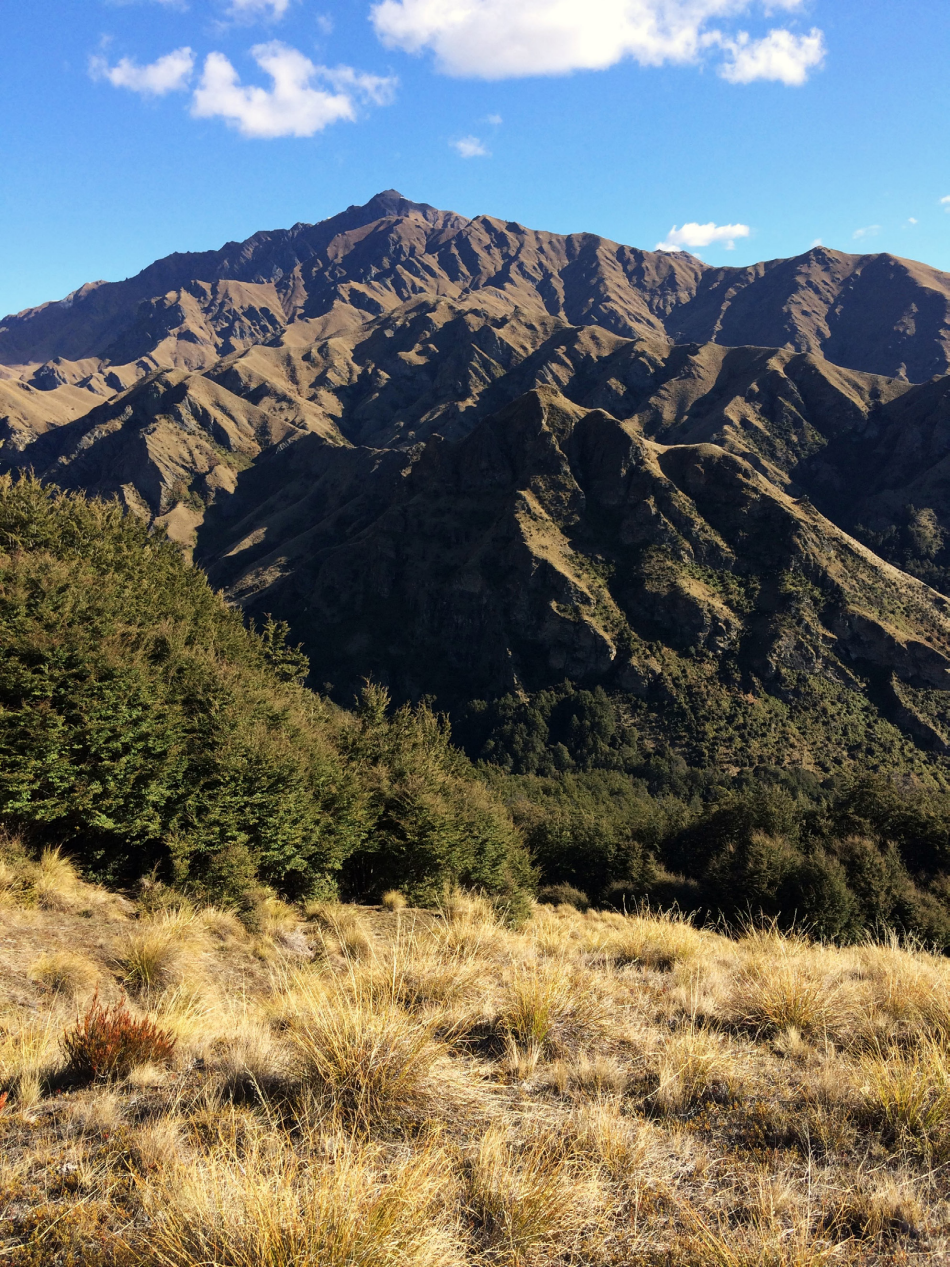
The boundary of a *Nothofagus cliffortioides* remnant forest in the Motatapu Valley, southern New Zealand. These boundaries tend to be stable over decadal time scales. Extensive areas of *Nothofagus* forest have been converted into grassland across New Zealand and a lack of ectomycorrhizal symbionts in grassland soils may be limiting natural forest recovery. This study investigates whether trophic facilitation using dual‐mycorrhizal shrubs, such as *Leptospermum*, could help establish an effective ectomycorrhizal community and promote remnant forest expansion.

*Nothofagus* (southern beech, Nothofagaceae; Govaerts (2022)) are an important group of southern hemisphere canopy trees in southern South America, eastern Australia, New Guinea, New Caledonia, and New Zealand (Veblen et al., 1996). In New Zealand, *Nothofagus* historically dominated forests over both main islands but were especially expansive in South Island (Wardle, 1984). Since human arrival in the 13th century, the extent of *Nothofagus* forest has been reduced through a combination of burning, farming, and forestry (Hall & McGlone, 2006). *Nothofagus* forests are still extensive in the western and southern parts of South Island, but in deforested areas, *Nothofagus* has largely been replaced by native or introduced grasslands and invasive wilding conifer forests (Froude, 2011). Natural *Nothofagus* recovery and spread from forest remnants is typically very slow, with boundaries often remaining stable for decades (Figure 1; Haase, 1989; Rogers, 1989; Wardle, 1980). Efforts to restore *Nothofagus* forests are increasing (van Galen et al., 2021) but often report variable or low success, especially in dryland areas in central and eastern South Island (Acevedo et al., 2019; Davis et al., 1997; Ledgard & Davis, 2004; Urretavizcaya et al., 2018; van Galen et al., 2022). A recent global meta‐analysis identified several factors that were important for *Nothofagus* restoration, including the provisioning of shelter and removal of grass competition (van Galen et al., 2021). However, that review also noted a surprising lack of research into the importance of ectomycorrhizal fungi during restoration (van Galen et al., 2021).

*Nothofagus* species form effectively obligate symbiotic relationships with ectomycorrhizal fungi, and it is likely that *Nothofagus* distributions are restricted to some extent by the availability of their fungal symbionts (Baylis, 1980; Dickie et al., 2012). Most ectomycorrhizal fungal species cannot survive without a host (Smith & Read, 2008), so deforested areas that lack host trees are typically devoid of appropriate mycorrhizal fungi. Although spore dispersal can be effective over short‐to‐medium distances (Peay et al., 2012), the absence of ectomycorrhizal fungi in soil may be a barrier to natural *Nothofagus* spread into suchareas and may be responsible for the poor success of many *Nothofagus* restoration projects (Dickie et al., 2012; van Galen et al., 2021). The only other common native ectomycorrhizal plants in New Zealand are pioneer shrubs and trees in the genera *Leptospermum* and *Kunzea* (Myrtaceae). Both *Leptospermum* and *Kunzea* are dual mycorrhizal and can form symbiotic relationships with arbuscular and ectomycorrhizal fungi (McKenzie et al., 2006). It has been hypothesized that *Leptospermum* and *Kunzea* species could be established in deforested areas where they can associate with arbuscular symbionts, and act as sinks for wind‐born ectomycorrhizal spores (Peay et al., 2012), enriching the ectomycorrhizal community over time and facilitating subsequent *Nothofagus* establishment (Burrows & Lord, 1993; Davis et al., 2013; Weijtmans et al., 2007). This could allow restoration projects to make use of natural trophic facilitation to improve outcomes for later successional trees.

The extent to which *Nothofagus* can form effective associations with the ectomycorrhizal fungi from *Leptospermum* and *Kunzea* soil is unknown. Ectomycorrhizal fungal species often show some level of specificity toward certain host types (van der Heijden et al., 2015; van Galen, Orlovich, Lord, Nilsen, et al., 2023), and given that these hosts are distantly related (order Fagales and order Myrtales), some divergence in symbiont compatibility might be expected. Past studies have found some shared fungal species between *Nothofagus* and *Kunzea* forests using environmental DNA (Teasdale et al., 2013), but surveys of fruiting bodies indicate that fungal communities of *Nothofagus* and *Leptospermum*/*Kunzea* forests can be highly divergent (McKenzie et al., 2000, 2006). Different fungal linages can also provide quite different functional services, for example, their capacity for water transport under drought conditions (Ruiz‐Lozano & Azcón, 1995). These functional differences could influence the effectiveness of *Leptospermum*/*Kunzea* fungal communities on *Nothofagus* establishment in dryland areas. Finally, in some cases, it has been shown that ectomycorrhizal fungi can form connections with a secondary non‐typical host if the primary host is also present (Pérez‐Pazos et al., 2021), so it is possible that growing *Nothofagus* and *Leptospermum*/*Kunzea* plants together could assist in the formation of associations with non‐typical fungal species.

Here, we use a pot experiment to test the hypothesis that *Leptospermum* can facilitate *Nothofagus* establishment by providing access to appropriate ectomycorrhizal fungi. We grew seedlings of *Nothofagus cliffortioides* (mountain beech, syn. *Fuscospora cliffortioides*; Heenan and Smissen (2013)) and *Leptospermum scoparium* (mānuka) in monoculture and mixed pairs with access to *Nothofagus*‐associated fungi, *Leptospermum*‐associated fungi, or both. We aimed to (1) characterize and compare the ectomycorrhizal community composition in *Nothofagus* and *Leptospermum* soil, (2) test whether root colonization, seedling biomass, and survival during drought stress differ when seedlings are grown in their “home” soil compared to “away” soil, and (3) test whether the presence of the primary host (“home” soil taxa) promoted the formation of mycorrhizal connections in the alternative host (“away” soil taxa) in mixed species plantings. These results will help to evaluate the potential for trophic facilitation to benefit forest restoration by determining the capacity for *Leptospermum‐*associated ectomycorrhizal fungi to facilitate the establishment of *Nothofagus*.

## 2 METHODS

### 2.1 Raising seedlings

*Nothofagus cliffortioides* (hereafter *Nothofagus*) and *Leptospermum scoparium* (hereafter *Leptospermum*) seeds were collected between January and April 2019 from natural forest and shrubland patches within a ~13 × 5 km area of the Motatapu Valley, New Zealand (−44.752° S, 168.910° E). *Nothofagus* seeds were stratified at 4°C for 3 months before sowing to stimulate germination (Wardle, 1984). *Leptospermum* capsules were stored at 20–30°C until they opened and released seeds, after which seeds were stored at room temperature until sowing. Seeds were sowed in June 2019 in trays containing 50% commercial potting mix and 50% coarse sand. Trays were kept in a glasshouse with a temperature range 20–25°C under natural light and watered regularly.

### 2.2 Experimental setup

#### 2.2.1 Experimental design

We conducted mycorrhizal bioassays by planting pairs of seedlings in different soil mixtures containing *Nothofagus* and/or *Leptospermum* fungal inoculum (Table 1; see further details below). Seedlings were grown in monoculture pairs (i.e., two *Nothofagus* or two *Leptospermum*) and mixed pairs. The experiment was established in September 2019 (3 months after sowing seeds) by transplanting seedlings into 32 × 13 × 5 cm trays containing soil treatments, with 12 seedlings (six pairs) per tray. Five replicated trays were planted for each combination of the three seedling pair types (*Nothofagus* monoculture, *Leptospermum* monoculture and mixed) and four soil treatments (*Nothofagus* inoculum “N”, *Leptospermum* inoculum “L”, *Nothofagus* and *Leptospermum* inoculum “NL”, and control “C”, Table 1). This resulted in 60 total trays containing 360 pairs of seedlings, with 30 replicate pairs of seedlings per treatment combination. Trays were kept in the glasshouse where seedlings were initially raised for 5 months.

**TABLE 1:** Details of the soil mixture treatments.

Treatment	Treatment code	Mixture
*Nothofagus* inoculum	N	25% unsterilized *Nothofagus* soil, 25% sterilized *Leptospermum* soil, and 50% sand
*Leptospermum* inoculum	L	25% sterilized *Nothofagus* soil, 25% unsterilized *Leptospermum* soil, and 50% sand
Dual inoculum	NL	25% unsterilized *Nothofagus* soil, 25% unsterilized *Leptospermum* soil, and 50% sand
Control	C	25% sterilized *Nothofagus* soil, 25% sterilized *Leptospermum* soil, and 50% sand

*Note*: Soil mixtures were created with equal parts of *Nothofagus* and *Leptospermum* soil with the sterilized/unsterilized status of the soil changed to introduce inoculum.

At the end of January 2020, seedlings were removed from the glasshouse and transplanted in their existing pairs into 1 L plastic pots containing freshly collected soil of the same mixtures (24–30 pots per treatment due to some seedling losses in trays, 341 pots in total). Pots were placed outside on raised pallets with weed mats underneath to allow roots to air prune before reaching the ground. A randomized block design with soil mixture treatments kept in separate blocks (two blocks per soil treatment, eight blocks in total) was used to reduce contamination between soils during watering. Host species combinations were randomized within each block. Because the experiment was conducted outdoors seedlings were potentially exposed to fungal spores in the air. However, all seedlings were exposed equally.

#### 2.2.2 Creating soil treatments

To create the soil medium for the bioassays, we collected the O horizon and the top of the A horizon from three pure *Nothofagus* and three pure *Leptospermum* forest patches within the Motatapu Valley near where seeds were collected. These horizons (often referred to as duff) harbor a large amount of ectomycorrhizal hyphal material (Genney et al., 2006). Soil from the same forest type was mixed, sieved to remove large debris, and divided in half. One‐half each of the *Nothofagus* and *Leptospermum* soil was sterilized to devitalize ectomycorrhizal fungal spores and hyphae by drying in thin layers at 60°C for 4 days. This temperature was chosen to render ectomycorrhizal fungal spores and hyphae inviable (Kipfer et al., 2010; Neary et al., 1999) without the risk of increasing available soil nutrients that can occur under higher temperatures (Dietrich et al., 2020). The remaining soil was stored at field moisture levels at 4°C.

All soil treatment mixtures (for both the initial trays and 1 L pots) were created by mixing equal parts *Nothofagus* and *Leptospermum* soil so that the soil structure, nutrients and other components were kept as similar as possible between treatments. The four treatments were created by altering the combinations of sterilized and unsterilized components to manipulate which ectomycorrhizal fungal communities were available to seedlings (Table 1). After mixing, an equivalent volume of coarse sand was added to each soil mixture so that seedlings were grown in a mix of 50% field soil and 50% sand.

### 2.3 Ectomycorrhizal community composition

Ectomycorrhizal fungal communities in pots were sampled using 4 × 4 cm hyphal ingrowth bags constructed from 50 μm nylon mesh and filled with 5 g of acid‐washed silica sand. Ingrowth bags largely select for active ectomycorrhizal mycelia growing in the soil (Bastias et al., 2006; van Galen, Orlovich, Lord, Bohorquez, et al., 2023; Wallander et al., 2001), and were chosen because sampling active hyphae provides an indication of the fungal species that have formed ectomycorrhizae with plants without the need to destructively sample seedlings. One disadvantage of this approach is that some taxa can show a preference for the substrate in the bag causing soil and bag communities to diverge, however, this effect is reduced with incubation times over 75 days (Hagenbo et al., 2018). One ingrowth bag was buried in four randomly selected pots for each treatment (soil mixture × host species combination) in February 2020, for a total of 48 samples. Bags were buried vertically 6 cm deep between the two seedlings in each pot. Ingrowth bags were recovered from the pots after 154 days and frozen at −20°C until DNA extraction.

Prior to DNA extraction, bags were thawed and their contents scraped into 50 mL falcon tubes. Hyphae were separated from the sand by adding 3 mL of autoclaved ultrapure water, vortexing, and allowing the mixture to settle for 15 min. The liquid and hyphae on top of the sand were then transferred to a 2 mL tube using a cut 1000 μL pipette tip, centrifuged to pellet the hyphae, and all liquid removed. To lyse cells prior to DNA extraction, between five and ten 2.3 mm zirconium beads were added to each tube, along with 400 μL of Qiagen DNeasy Plant Mini‐Kit DNA extraction buffer AP1 and 4 μL of RNase A (concentration 100 mg/mL), and tubes were shaken on a bead beater (Biospec Mini‐Beadbeater‐96) for three bursts of 30 s. Samples were then placed in a heated block at 60°C for 10 min, and the DNA extraction process was continued using the Qiagen DNeasy Plant Mini Kit following the manufacturer's instructions.

We undertook PCR amplification of the internal transcribed spacer 2 (ITS2) region using the forward primer fITS7 (Ihrmark et al., 2012) and reverse primer ITS4 (White et al., 1990). Each PCR reaction included 12.5 μL of KAPA HiFi HotStart Ready Mix, 10 pmol of each primer, 1 μL of 50 mg/mL bovine serum albumin, 9.5 μL of autoclaved ultrapure water, and 1 μL of DNA extract. The amplification protocol was 95°C for 3 min, followed by 35 cycles of 95°C for 30 s, 57°C for 30 s, 72°C for 45 s, and a final elongation of 72°C for 5 min. Negative controls were included for all PCR runs to ensure no contamination occurred. DNA amplicons were subsequently purified using the Agencourt AMPure XP PCR Purification kit following the manufacturer's protocol, diluted to a concentration of 5 ng/μL, and sent to Massey Genome Service Facility (Massey University, New Zealand) for second‐round PCR, library preparation, and Illumina MiSeq sequencing on a 2 × 250 bp paired‐end run.

**FIGURE A1:**
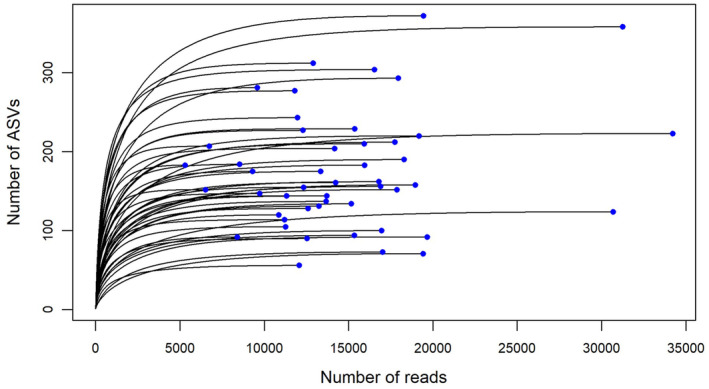
Rarefaction curves for each of the 48 hyphal ingrowth bags showing the number of total ASVs detected (including non‐ectomycorrhizal ASVs) with increasing sequencing read depth. Blue points show the total number of reads for each sample.

Raw sequences were processed using the DADA2 pipeline (Callahan et al., 2016) in R version 3.6.2 (R Development Core Team 2019). Primers were removed using “cutadapt” version 1.14 (Martin, 2011) with up to 20% mismatches allowed. Reads were then trimmed and filtered using the “filterAndTrim” function. Reads were truncated at the first instance of a quality score representing two or more expected errors. Reads shorter than 50 bp after truncation were discarded. After processing, 2905 amplicon sequence variants (ASVs) were present, and each one was assigned a putative taxonomic identification using the “assignTaxonomy” function with the UNITE database version 8.3 general release FASTA file (Kõljalg et al., 2020; Nilsson et al., 2018) as the reference database (including species hypotheses represented by single sequences). The minimum bootstrap confidence for assigning a taxonomic level was set at the default of 50. ASVs were then classified into guilds using the FUNGuild database (Nguyen et al., 2016), and all those assigned as ectomycorrhizal with a “highly probable” confidence ranking were retained for analysis. Those identified as *Amanita* species were also retained due to the known association between *Amanita* and *Nothofagus* in New Zealand (McKenzie et al., 2000). In total, 100 ASVs were classified as ectomycorrhizal and used in the analyses. Rarefaction curves showed that the sequencing read depth was sufficient to detect the vast majority of ASVs present in each sample (Figure A1), and so read counts were standardized by dividing the number of reads of each ectomycorrhizal ASV by the total number of ectomycorrhizal reads in the sample (McKnight et al., 2019).

### 2.4 Root colonization

**FIGURE B1:**
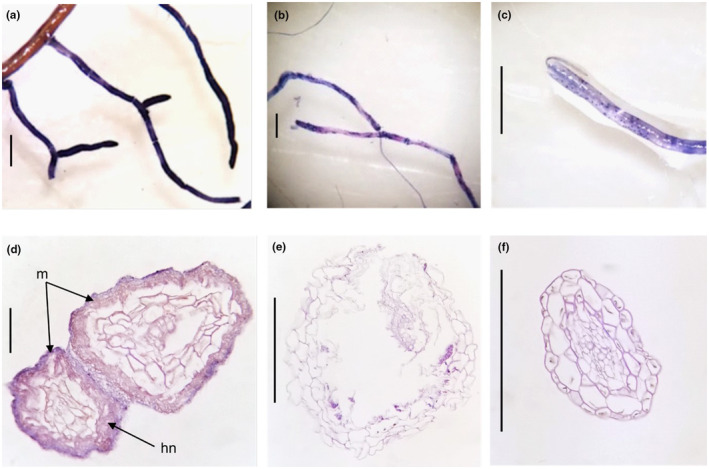
Stained *Leptospermum* root tips under a dissecting microscope (a–c) and corresponding cross sections under a compound microscope at 40 × magnification (d–f). (d) Shows a mantle sheath (m) and Hartig Net (hn) present in the roots of (a). No ectomycorrhizal structures were visible in cross sections of the roots of (b and c) (e and f). Scales bars are approximately 1 mm in A to C and 50 μm in D to F.

Sixty‐four seedlings were selected using stratified random sampling to assess ectomycorrhizal colonization across all treatments in August 2020 (11 months after establishment). Seedlings were selected randomly from each of the 12 soil mixture × host species combinations, including five *Nothofagus* and five *Leptospermum* seedlings from monoculture combinations and three *Nothofagus* and three *Leptospermum* seedlings from mixed combinations for each soil mixture. A 500 mg root subsample (fresh weight) was taken from each seedling. All root samples were cleaned in water until free of soil particles, and stained with Trypan Blue following a modified version of the staining protocol from Brundrett et al. (1996). Large root samples were firstly subsampled, fine roots (<1 mm thick) were selected, and samples were rinsed three times in purified water. Samples were then covered with 5% KOH and cleared by heating in a microwave until they began to soften (Dalpé & Séguin, 2013). Samples were drained and rinsed with purified water and bleached for 10 min with either 5% HCl for *Leptospermum* roots or 0.5% v/v H_2_O_2_ for *Nothofagus* roots as recommended by Nylund et al. (1982) and Brundrett and Kendrick (1990) for viewing ectomycorrhizae (Brundrett et al., 1996). Roots were covered with 0.05% w/v Trypan Blue in lactoglycerol (1:1:1 lactic acid, glycerol, and purified water) and placed in a 65°C water bath for 10 min. Roots were then removed from the staining solution and placed in Petri dishes containing lactoglycerol without the stain. All samples were examined under a dissecting microscope and a random subsample of approximately 300 root tips was scored as ectomycorrhizal (dark purple staining and sometimes swollen) or non‐ectomycorrhizal (no thickening and little staining; see Figure B1) to calculate the percentage of tips colonized.

### 2.5 Plant performance

#### 2.5.1 Biomass

Plant performance was assessed by measuring the dry‐weight biomass from the same 64 seedlings used to quantify ectomycorrhizal colonization. Seedlings were gently rinsed in water to remove soil, then shoots and remaining roots were dried for 3 days at 70°C and weighed. To account for the 500 mg of root material taken for ectomycorrhizal quantification, 500 mg was dried and weighed separately for a *Nothofagus* and *Leptospermum* seedling from each treatment combination, and the average of those for each species was added to the weights of the respective seedlings.

#### 2.5.2 Drought tolerance

After the ectomycorrhizal and biomass sampling had taken place, the remaining seedlings experienced an unplanned but significant drought event. The watering system failed for at least 1 week during unseasonably warm weather in October 2020, when daytime maximum temperatures reached up to 23.3°C and less than 2 mm of rain fell. As a result, many seedlings succumbed to drought stress, and we scored survival to determine if the different treatments provided different levels of protection against drought.

### 2.6 Statistical analysis

All statistical analyses were performed in R version 4.2.1 (R Core Team, 2022) and all plots were generated using the “ggplot2” package version 3.3.6 (Wickham, 2016).

To examine differences between the ectomycorrhizal fungal communities of different soil mixtures and host species combinations, we performed a two‐dimensional non‐metric multidimensional scaling (NMDS) ordination of ectomycorrhizal ASVs based on Bray–Curtis dissimilarity using the “metaMDS” function from the “vegan” package (Oksanen et al., 2020). Dissimilarity was calculated based on a presence/absence occurrence matrix of the 100 ectomycorrhizal ASVs in the 48 samples. Analysis of similarities (ANOSIM) using the “anosim” function was performed to test for differences in ordination scores among soil mixtures and host species combinations.

We examined differences in ASV richness among soil mixtures and host species combinations using linear models with square‐root‐transformed richness data to improve normality. We tested whether an interaction between soil and species combination treatments existed by comparing models with and without the interaction term using the “anova” function to conduct likelihood ratio tests. There was no significant difference between models (*p* = .80), so the simpler model without the interaction was used. A Tukey test was performed to calculate pairwise comparisons between treatment levels using the “glht” function from the “multcomp” package (Hothorn et al., 2008).

We also used linear models to examine the effect of soil mixtures and host species combinations on (a) ectomycorrhizal root colonization and (b) seedling biomass. Colonization and biomass were measured at the seedling level rather than the pot level, which introduces seedling identity (*Nothofagus* or *Leptospermum*) as an additional variable. However, the data did not provide enough power to support including a three‐way interaction term among soil mixture, host species combination (monoculture or mixed) and seedling identity (*Nothofagus* or *Leptospermum*). Therefore, we ran two groups of models: (1) one‐way ANOVAs to test overall differences in colonization and biomass between *Nothofagus* and *Leptospermum* seedlings, and (2) separate linear models for *Nothofagus* and *Leptospermum* seedlings, each testing two‐way interactions between soil mixture and host species combination on colonization and biomass. Root colonization data were untransformed, but seedling biomass was log transformed to improve normality. As with ASV richness, we compared models with and without the interaction term with likelihood ratio tests. The interaction term significantly improved both the colonization and biomass models for *Nothofagus* seedlings and the colonization model for *Leptospermum* seedlings, (*p* < .023), but not the *Leptospermum* biomass model (*p* = .66), and so the interaction term was dropped from the *Leptospermum* biomass model. Tukey tests were used to conduct pairwise comparisons as described above.

To investigate whether variation in seedling biomass could be due to different root colonization levels or ectomycorrhizal ASV richness, we performed linear mixed‐effects models for (a) *Nothofagus* and (b) *Leptospermum* seedlings testing the effect of colonization and richness on log‐transformed biomass. We included soil mixture as a random factor to allow intercepts to vary between soil types. Colonization was measured using the same 64 seedlings randomly selected to calculate biomass, but ASV richness was measured in a different set of 48 randomly selected pots (four pots of each soil mixture × host species combination treatment). The selected pots within each soil mixture × host species combination were randomly distributed across two blocks (eight blocks in total, see Section 2.2). Therefore, in the biomass × richness contrast, we used the average richness value from the pots of the treatment and block that the corresponding biomass values came from. We used the “lmer” function from the “lme4” R package (Bates et al., 2015) to run the models with Tukey tests to conduct pairwise comparisons. We assessed the statistical significance of each term (colonization and richness) by performing likelihood ratio tests with the “anova” function to compare the full model to a model with the term removed. We used the “ggpredict” function from the “ggeffects” package (Lüdecke, 2018) to extract prediction data from the full model for plotting trend lines.

We examined the effects of soil mixture and host species combination on survival following drought for (a) *Nothofagus* and (b) *Leptospermum* seedlings using binomial models with the “glm” function and Tukey tests to conduct pairwise comparisons. We also included biomass as a covariate because greater transpiration by larger seedlings likely influenced their susceptibility to drought treatment. As described above, we tested whether including the interaction between soil mixture and species combination improved the models using likelihood ratio tests. There was no significant interaction for either the *Nothofagus* (*p* = .14) or *Leptospermum* (*p* = .41) models, and so the interaction term was removed. In addition to examining treatment effects on survival, we also ran models to test whether any variation in survival could be attributed to ectomycorrhizal root colonization levels or ASV richness. We ran mixed‐effects binomial models using the “glmer” function separately for *Nothofagus* and *Leptospermum* survival with colonization and richness as predictor variables. Because colonization and richness were not measured for every seedling, we assigned each seedling the average colonization and richness values collected from the appropriate soil mixture × host species combination × block treatment (as described in the previous paragraph). We also included the average biomass from the treatment combination as a covariate, and the soil mixture as a random factor to allow intercepts to vary between soil treatments.

## 3 RESULTS

Metabarcoding of hyphal ingrowth bag samples returned 716,156 sequence reads belonging to 2905 ASVs, of which 242,483 reads (33.9%) of 100 ASVs (3.4%) were classified as ectomycorrhizal. Ectomycorrhizal ASVs were putatively identified as belonging to 17 genera, with the vast majority from the phylum Basidiomycota (99 ASVs) and only one ASV from Ascomycota.

### 3.1 Ectomycorrhizal community composition

#### 3.1.1 Soil mixtures

**FIGURE 2:**
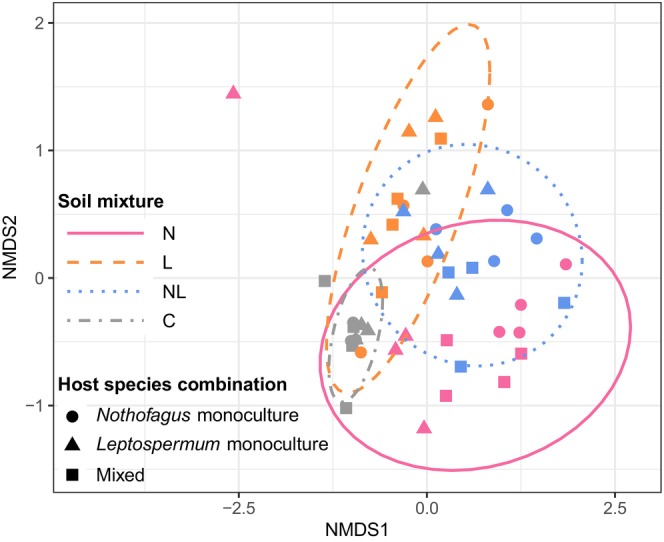
Ordination by non‐metric multidimensional scaling of ectomycorrhizal fungal amplicon sequence variants (ASVs) based on Bray–Curtis dissimilarity calculated using presence/absence ASV data. Ellipses show 95% confidence regions of groups based on a multivariate *t*‐distribution. Stress = 0.12. C = control; L = *Leptospermum*‐inoculated soil; N = *Nothofagus*‐inoculated soil; NL = dual‐inoculated soil.

**FIGURE 3:**
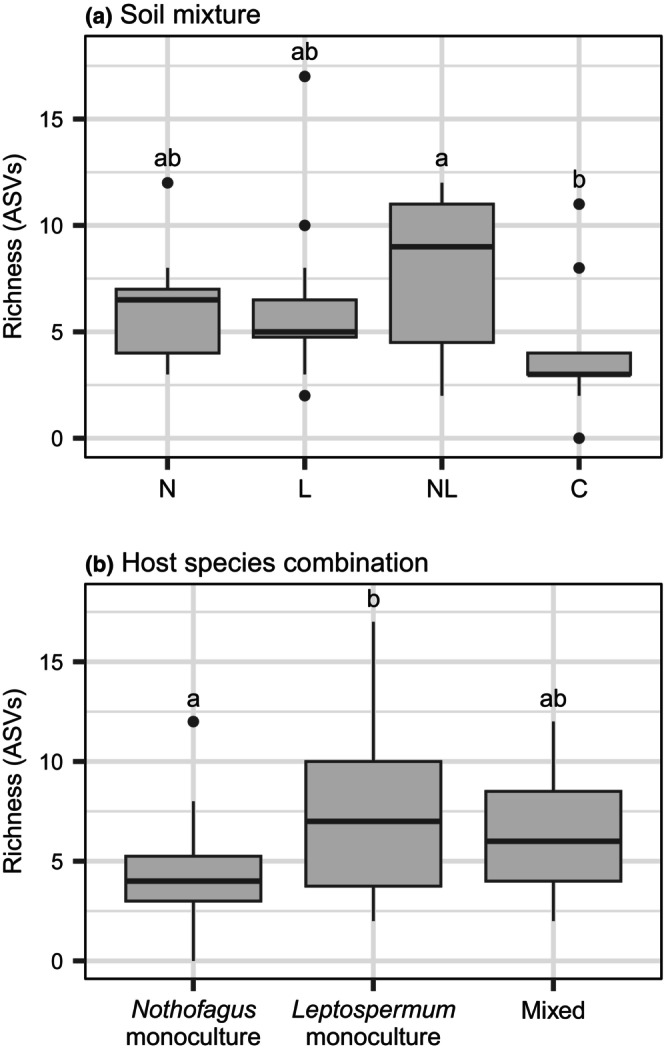
Ectomycorrhizal fungal richness (number of ASVs) of soil mixtures and host species combinations. There was no interaction between the two treatments so the results are presented separately for each. Boxes with the same letter within each panel are not significantly different. *n* = 12 for each soil mixture in (a) and 16 for each host species combination in (b). C = control; L = *Leptospermum*‐inoculated soil; N = *Nothofagus*‐inoculated soil; NL = dual‐inoculated soil.

**FIGURE 4:**
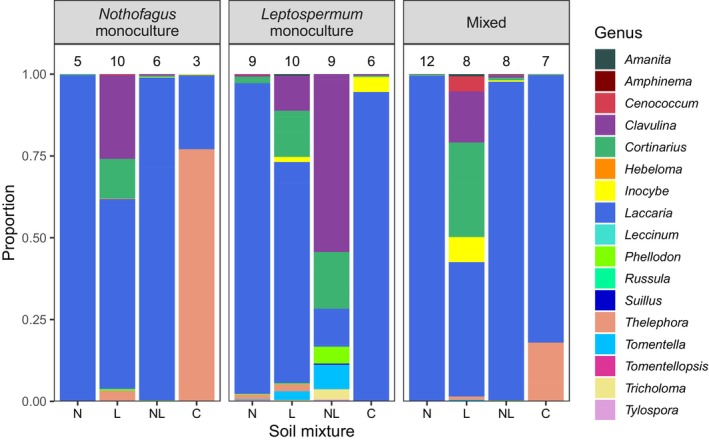
Relative proportion (based on normalized sequence read abundance) of ectomycorrhizal fungal genera in each treatment. Numbers above bars indicate the total number of genera present. *n* = 4 pots/ingrowth bags for all bars. C = control; L = *Leptospermum*‐inoculated soil; N = *Nothofagus*‐inoculated soil; NL = dual‐inoculated soil.

The ordination and ANOSIM revealed differences in ectomycorrhizal fungal community composition among soil mixtures (*p* = .001, *R* = .455), with the *Nothofagus‐* (N) and *Leptospermum‐*inoculated (L) communities forming divergent clusters and the dual‐inoculated mixture (NL) intermediate to these (Figure 2). ASV richness was similar in *Nothofagus‐* and *Leptospermum‐*inoculated soil, and there was a trend of greater richness in the dual‐inoculated soil, although this trend was not statistically significant (*p* > .78, Figure 3a). Regardless of the host species combination (i.e., monocultures or mixed), fungal sequence reads from *Nothofagus‐*inoculated soil (N) were mostly dominated by *Laccaria* sequences, whereas *Leptospermum*‐inoculated soil (L) contained a more even community that included not only *Laccaria* but also many *Cortinarius*, *Clavulina*, *Inocybe* and *Cenococcum* sequences (Figure 4). The control soil mixture (C) still contained ectomycorrhizal fungal ASVs, although ASV richness (Figure 3a) and the number of genera present (Figure 4) were less than in the other treatments.

Of the 27 most commonly detected ASVs, 11 were also detected in the control (Table 2) and may therefore have originated from external spore sources or have survived the heat sterilization treatment (see Section 4). Of the remaining 16, six were only detected when *Nothofagus* inoculum was present (treatments N or NL; belonging to the genera *Cortinarius*, *Laccaria*, *Clavulina*, and *Phellodon*), two were only detected when *Leptospermum* inoculum was present (L or NL; *Cortinarius* and *Inocybe*), and eight were detected in both inoculum types (*Cortinarius*, *Phellodon*, *Cenococcum*, *Tylospora*, and *Tomentella*; Table 2). However, the small number of samples collected from each treatment combination (12 from each soil inoculum mixture, 4 per soil mixture × species combination) means that ASVs may have remained undetected in some treatments, and so it is not possible to fully quantify the number of unique and shared ASVs across treatment types.

**TABLE 2:** Prevalence (number of samples) of ASVs detected in at least three samples (total samples = 48, four per treatment combination).

ASV ID	Putative species ID	Total # samples	*Nothofagus* inoculum			*Leptospermum* inoculum			Dual inoculum			Control			Soil type specificity
			N mono	L mono	Mixed	N mono	L mono	Mixed	N mono	L mono	Mixed	N mono	L mono	Mixed	
asv_2	*Laccaria* sp.	25		2		3	2	3	1	2	2	3	4	3	
asv_7	*Thelephora terrestris*	19		3	2	1	1	1		1		3	3	4	
asv_186	*Clavulina* sp.	18				3	3	2	3	4	2		1		
asv_831	*Phellodon* sp.	17	3	2	2	1	1		2	3	3				NL
asv_1	*Laccaria* sp.	16	4		4				3	1	4				N
asv_201	*Cortinarius* sp.	16			1	1	4	4	1	3	1		1		
asv_1407	*Clavulina* sp.	8	1	2	1				1	2	1				N
asv_5	*Cenococcum* sp.	7		1	1	1		1	1		2				NL
asv_316	*Inocybe leptospermi*	7				1	1	1	1		2		1		
asv_21	*Laccaria* sp.	6				1						2	1	2	
asv_1631	*Cortinarius paraxanthus*	6					2	1	3						L
asv_77	*Amanita muscaria*	5			1		1				2		1		
asv_1243	*Cortinarius* sp.	5				1				1	2		1		
asv_1519	*Phellodon* sp.	5	1		2				1	1					N
asv_2197	*Tylospora* sp.	5		1		1				1	2				NL?
asv_853	*Laccaria* sp.	4	2		1						1				N
asv_1415	*Cortinarius pholiotellus*	4					1	1		1			1		
asv_2134	*Cortinarius* sp.	4	1		1					1			1		
asv_2236	*Cortinarius* sp.	4		1			1			1	1				NL?
asv_4	*Cortinarius vitreopileatus*	3			1		2								NL?
asv_9	*Cortinarius subcastanellus*	3			1		1				1				NL?
asv_26	*Cortinarius anisodorus*	3	2					1							NL?
asv_396	*Cortinarius* sp.	3		1	2										N
asv_1323	*Cortinarius* sp.	3	1	1	1										N
asv_2110	*Cortinarius waiporianus*	3							1	1				1	
asv_2503	*Inocybe leucotaenia*	3					2	1							L
asv_2974	*Tomentella* sp.	3		1			2								NL?

*Note*: The final column indicates if ASVs were only detected when *Nothofagus* inoculum was unsterilized (N), only when *Leptospermum* inoculum was unsterilized (L), or in both (NL). These classifications are only made for ASVs not detected in the control. Question marks show uncertain associations when ASVs were detected in only one sample of an inoculum type. Note that the limited number of samples per treatment combination could mean that some ASVs remained undetected.

Abbreviations: L, *Leptospermum*; “mono” = monoculture; N, *Nothofagus*.

#### 3.1.2 Host species combinations

Soil fungal communities did not statistically differ among *Nothofagus* monoculture, *Leptospermum* monoculture, and mixed host combinations (ANOSIM: *p* = .27, *R* = .016; Figure 2). However, *Leptospermum* monoculture pots had significantly higher ASV richness than *Nothofagus* monoculture pots (*p* = .04), with mixed seedling pots containing intermediate richness (Figure 3b). There was no interaction effect between soil mixture and host species combination on ASV richness (*p* = .80), indicating that these richness levels were the same regardless of whether seedlings were grown with access to “home” or “away” soil fungi. However, there were differences in the fungal genera detected by ingrowth bags between “home” and “away” treatments (Figure 4). For example, when *Nothofagus* seedlings were grown as monocultures in their “home” soil (N), the vast majority of sequences detected belonged to *Laccaria* species, whereas when grown in the “away” *Leptospermum*‐inoculated soil (L), sequences belonging to *Clavulina* and *Cortinarius* were also abundant. Similar differences were observed between *Leptospermum* monoculture pots containing “home” (L) and “away” (N) soils, providing further evidence that soil inoculum type has a stronger influence on fungal community composition than the host species present. When given access to all types of fungi in the dual‐inoculation treatment (NL), the fungal communities detected tended to be more similar to those from the “home” soil treatment (Figure 4). For example, dual‐inoculated communities of *Nothofagus* monoculture pots were more similar to *Nothofagus*‐inoculated communities, whereas dual‐inoculated *Leptospermum* monoculture pots were more similar to *Leptospermum*‐inoculated communities (Figure 4). However, mixed soil inoculum in mixed species pots developed communities more like *Nothofagus* home soil (Figure 4). This may indicate that *Nothofagus* is relatively competitive in the soil combination provided (Table 1).

### 3.2 Ectomycorrhizal colonization and plant performance

**FIGURE 5:**
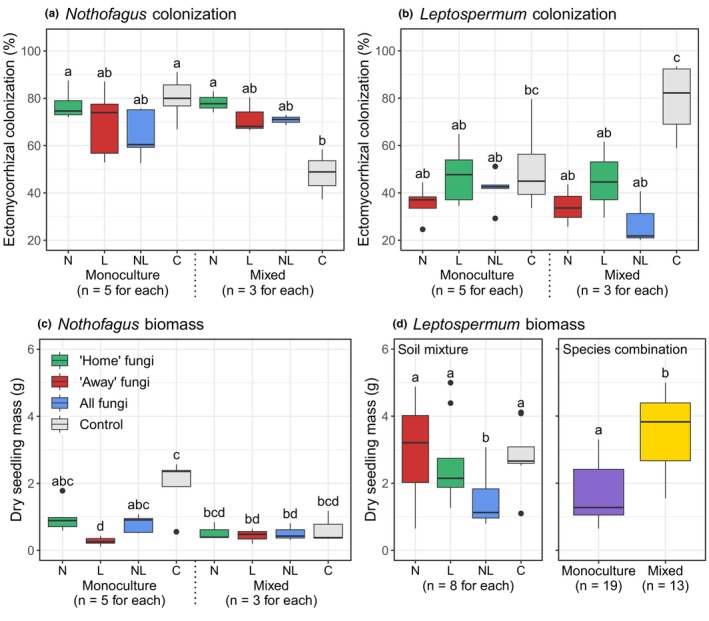
Ectomycorrhizal colonization and dry seedling biomass of (a, b) *Nothofagus* seedlings and (c, d) *Leptospermum* seedlings. There was an interaction between soil mixture and host species combination for *Nothofagus* models and the *Leptospermum* colonization model, and so results are presented for all combinations of treatment levels in (a–c). There was no interaction in the *Leptospermum* biomass model, so the effect of soil mixture and host species combination are shown separately in (d). Boxes within each panel that share a letter are not significantly different. C = control; L = *Leptospermum*‐inoculated soil; N = *Nothofagus*‐inoculated soil; NL = dual‐inoculated soil.

Ectomycorrhizal root colonization was significantly higher for *Nothofagus* seedlings than for *Leptospermum* seedlings (mean colonization = 70.8% and 45.8%, respectively; ANOVA: *F*_1,62_ = 52.76, *p* < .001). There were no significant differences in colonization between seedlings grown with access to “home”, “away”, or all (NL) soil fungi (*p* > .40), but there was a trend of higher colonization in “home” soil mixtures for both *Nothofagus* and *Leptospermum* seedlings (Figure 5a,b). Interestingly, colonization rates in the control soil mixture were higher than some of the inoculated soil mixtures for *Leptospermum* seedlings (Figure 5b) but were either similar or lower for *Nothofagus* seedlings (Figure 5a). Whether seedlings were grown in monoculture or mixed pairs did not affect colonization levels in most cases (Figure 5a,b), except for colonization of *Nothofagus* seedlings in the control soil mixture, where colonization was significantly lower (*p* < .001) in mixed host pots (Figure 5a).

**FIGURE 6:**
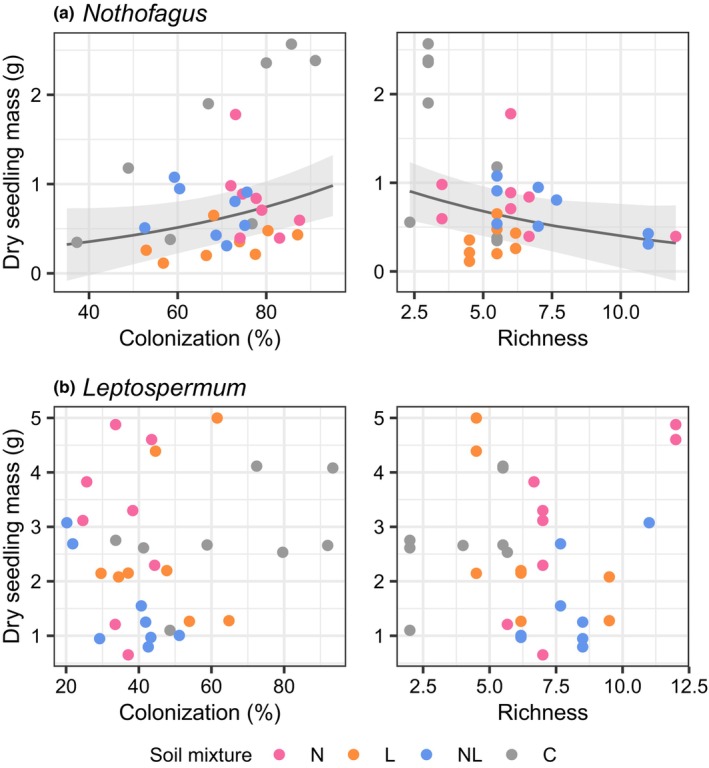
Relationships between dry seedling biomass and ectomycorrhizal root colonization and ASV richness for (a) *Nothofagus* seedlings and (b) *Leptospermum* seedlings. Trend lines show significant relationships and shading 1 SE. Soil mixture was included in models as a random effect and is shown as colors.

*Nothofagus* seedlings contained significantly less biomass than *Leptospermum* seedlings (mean = 0.83 and 2.47 g, respectively; ANOVA: *F*_1,62_ = 52.76, *p* < .001). Biomass of *Nothofagus* seedlings was significantly higher in the “home” soil fungal treatment (N) compared to the “away” treatment (L) when grown in monoculture pairs (*p* < .01), but not when grown in mixed pairs (*p* = .999; Figure 5c). There was no difference in biomass of *Leptospermum* seedlings grown in “home” (L) or “away” (N) soil treatments (*p* = .96), but interestingly, biomass was higher in both these soil treatments than the dual‐inoculated treatment (NL; *p* < .04; Figure 5d). *Leptospermum* seedlings grown in mixed pairs had greater biomass than those grown in monoculture pairs (*p* < .001; Figure 5d). For *Nothofagus*, biomass was positively related to colonization levels (*p* = .027) and negatively related to ASV richness (*p* = .035; Figure 6). Biomass was not significantly related to colonization or richness for *Leptospermum* (*p* = .74 and .48, respectively; Figure 6).

**FIGURE 7:**
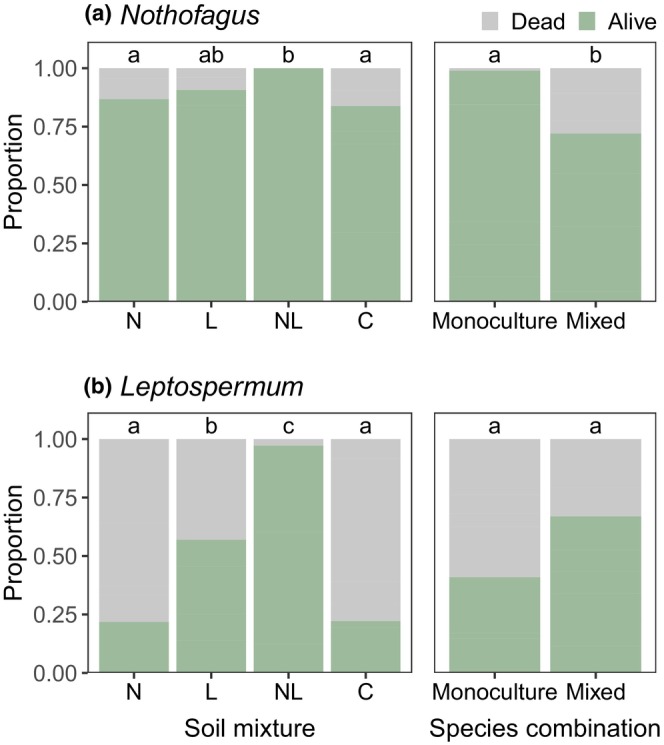
Seedling survival status in different treatments of (a) *Nothofagus* and (b) *Leptospermum* in response to drought stress. Seedling biomass was included within each model as a covariate. C = control; L = *Leptospermum*‐inoculated soil, N = *Nothofagus*‐inoculated soil, NL = dual‐inoculated soil.

When subjected to drought stress, 90.3% of *Nothofagus* seedlings survived but only 47.9% of *Leptospermum* seedlings survived. There was no interaction effect between soil mixture and species combination treatments on survival (*p* > .14). For *Leptospermum*, survival was significantly higher for “home” soil mixtures containing *Leptospermum* fungal inoculum (L and NL; *p* < .001), with survival in the dual‐inoculation treatment (NL) significantly higher than all other treatments (*p* < .001, Figure 7b). For *Nothofagus*, survival was high in all treatments but was significantly higher in the dual‐inoculation treatment compared to *Nothofagus*‐inoculated and control treatments (*p* = . 047 and .021, respectively; Figure 7a). Interestingly, *Nothofagus* survival was significantly higher when grown in monoculture pairs (*p* < .001), whereas survival of *Leptospermum* was greater when grown in mixed pairs (although differences were non‐significant, *p* = .062; Figure 7). This is likely due to an interaction between drought stress and biomass, where larger plants are more affected by drought due to increased transpiration. Given that *Leptospermum* seedlings were larger than *Nothofagus* seedlings (Figure 5), monoculture pots contained relatively lower biomass for *Nothofagus* seedlings, whereas mixed pots contained relatively lower biomass for *Leptospermum* seedlings. Examining the effect of ectomycorrhizal root colonization levels and ASV richness on survival (where each seedling was assigned the average colonization and richness levels from the appropriate treatment combination; see Section 2.6) showed that, for *Nothofagus* seedlings, survival was significantly improved by higher ectomycorrhizal root colonization levels (estimate = 0.05, *z* = 2.21, *p* = .028) but was marginally negatively related to ectomycorrhizal ASV richness (estimate = −0.21, *z* = −1.97, *p* = .049). For *Leptospermum* seedlings, survival was not significantly related to root colonization levels (estimate = 0.03, *z* = 1.63, *p* = .103) or ASV richness (estimate = 0.15, *z* = 1.78, *p* = .075).

### 3.3 Can leptospermum trophically facilitate Nothofagus establishment?

We compared the *Nothofagus* monoculture treatment when grown with access to “home” (N) versus “away” (L) soil fungi to determine the ability of *Nothofagus* seedlings to associate with *Leptospermum* fungal species. This showed that root colonization was similar (Figure 5a) but seedling biomass was slightly reduced (Figure 5c) in the “away” treatment. However, of the 10 ASVs detected in this treatment (considering those detected in at least three samples; Table 2), 7 were also detected in the control. This indicates that the high root colonization may have been the result of the environmental contaminants and not true *Leptospermum* fungal species. However, the three ASVs not detected in the control are likely to be *Leptospermum*‐associated species. Additionally, 50% of the ASVs in Table 2 not found in the control were detected in both soil types, indicating that generalist fungal species are present in *Leptospermum* soil which may facilitate *Nothofagus* establishment. However, further evidence is needed to determine the true extent to which *Nothofagus* can utilize *Leptospermum* fungal species in the absence of the environmental contaminants present in this experiment.

## 4 DISCUSSION

Although not entirely conclusive, our results are consistent with the notion that *Nothofagus* and *Leptospermum* may be able to share ectomycorrhizal symbionts to improve restoration success. The fact that half of the most prevalent ASVs not present in the controls were detected in both *Nothofagus* and *Leptospermum* soil inoculum types indicates the likely presence of generalist fungi that could aid trophic facilitation. However, we were unable to fully quantify the number of shared and unique ASVs present across treatment types, and several of the most prevalent ASVs were also present in the controls. This likely environmental contamination (see below) makes the sources of ectomycorrhizal colonization in some treatments ambiguous. Despite this ambiguity, ectomycorrhizal fungal communities clearly differed between *Nothofagus* and *Leptospermum* soil, both host species appear able to form connections with fungal ASVs from “away” soil inoculum, and there was evidence of a “home” soil inoculum advantage in some treatments. There was no evidence in mixed seedling treatments that the presence of the primary host (“home” soil plant species) improved colonization or performance of the alternative host (“away” soil plant species), although it is possible that colonization by the environmental contaminants may have masked subtle effects.

The control contamination may have originated from fungal propagules surviving the sterilization treatment or from airborne spores. Although, it is possible some fungal species survived, our 4‐day heat sterilization method was designed to minimize this risk. Airborne contamination was almost certainly an issue, and is a well‐documented problem in nursery and glasshouse studies working with ectomycorrhizal fungi (Marx & Bryan, 1969; Sanchez‐Zabala et al., 2013; Stottlemyer et al., 2008). Mature fruiting bodies matching the most common ASV in the control samples (*Laccaria* sp.; Table 2; confirmed with sanger sequencing) were found growing in a garden within meters of the experimental area. Additionally, the second most common ASV in the controls was *Thelephora terrestris*, a notorious experimental pest known for its global distribution, wide host range (Marx & Bryan, 1969; Smith & Read, 2008; Stottlemyer et al., 2008), and tendency to contaminate ectomycorrhizal pot experiments, even affecting studies conducted in high‐efficiency particulate air‐filtered chambers (Stottlemyer et al., 2008). The likely contamination in our experiment means that some treatment effects may be masked by the contaminants, and non‐significant results should be treated with caution. However, significant differences between treatments remain valid.

The uncertainty caused by the contamination of control samples affects our test of whether *Nothofagus* species can utilize ectomycorrhizal fungi from *Leptospermum* soil. On one hand, three of the most common fungi *not* found in the controls were detected in the *Nothofagus* monoculture growing with *Leptospermum* inoculum, indicating that *Nothofagus* can utilize fungi from this inoculum. However, because seven other ASVs detected in this treatment were present in the controls, we cannot determine what proportion of the ectomycorrhizal colonization and subsequent growth benefits (Figure 5a) resulted from fungal contamination versus ASVs from the *Leptospermum* inoculum. Interestingly, when *Leptospermum* monocultures were growing with *Nothofagus* inoculum, only 2 of 10 common ASVs were present in the controls, and 3 were specific to *Nothofagus* inoculum. These results indicate that *Leptospermum* may have lower symbiont specificity than *Nothofagus*, which is consistent with their ecological roles as early and late successional taxa, respectively (Burrell, 1965). It should also be noted that the growth responses observed in this pot experiment might differ under field conditions because of different taxa contributing to the environmental species pool.

That *Nothofagus* seedlings may be able to associate with fungal species from *Leptospermum* soil provides promise that trophic facilitation could be used to improve restoration. There is ample evidence that inoculating with mycorrhizal fungal species when planting trees can improve vegetation establishment and succession (Koziol et al., 2022; Maltz & Treseder, 2015; Neuenkamp et al., 2019), but ectomycorrhizal fungal species are difficult to culture and inoculation methods that are efficient to apply at large scales are not well developed (Brundrett et al., 1996). Our results indicate that natural trophic facilitation could be harnessed as a way of improving the soil microbiome required for late‐successional canopy trees. There is evidence from past studies that *Nothofagus* seedlings perform better when growing close to dual‐mycorrhizal shrubs like *Leptospermum* or *Kunzea* (Burrows & Lord, 1993; Davis et al., 2013; Tulod & Norton, 2020a), and Dickie et al. (2012) also observed high ectomycorrhizal colonization levels in *Nothofagus* seedlings grown in *Kunzea* soil. Our results add new evidence that the fungal communities associated with seedlings in *Leptospermum* stands are somewhat different from those in *Nothofagus* forest soil, but they potentially still form effective symbioses. In future research, it would be useful to employ root tip analysis to confirm the associations detected in our study using hyphal ingrowth bags. Further work is also needed in the absence of environmental contaminants to quantify the degree to which *Nothofagus* seedlings profit from colonization by fungal species from the *Leptospermum* community.

The benefits gained from trophic facilitation occurring throughout the successional process are also complemented by physical benefits, such as the shade and shelter that early‐successional species provide for later‐successional ones (Crouzeilles et al., 2017; Davis et al., 2013; Tulod & Norton, 2020a). Focusing restoration purely on the establishment of early‐successional plants like *Leptospermum* comes with risks because pioneer species can sometimes inhibit the development of more diverse later‐successional communities (Tulod & Norton, 2020b), or create fire‐prone early‐successional states that self‐perpetuate (Lord et al., 2022). However, by stimulating succession, trophic facilitation lowers such risks, allowing late‐successional plants to benefit from both shelter and the mycorrhizal symbionts available. For *Nothofagus*, the availability of shade and shelter is known to be a critical factor in seedling establishment and survival (Davis et al., 2013; Henríquez & Lusk, 2005; Tulod & Norton, 2020a; Valenzuela et al., 2016; van Galen et al., 2021, 2022), so a successional approach using dual‐mycorrhizal shrubs is likely to provide both trophic and non‐trophic benefits.

Our finding of a “home” soil advantage in some treatments suggests that fungal community composition could affect seedling performance in the field. *Nothofagus* seedlings had greater biomass in monoculture pots when inoculated with “home” *Nothofagus* fungi compared to “away” *Leptospermum* fungal communities, a response that has also been found in many arbuscular mycorrhizal fungal inoculation trials (Rúa et al., 2016). Interestingly, we also found that mixed seedlings growing in mixed inoculum tended to develop ectomycorrhizal communities more like *Nothofagus* home soil. This suggests that *Nothofagus* may be a more competitive host, at least in the soil conditions provided in this experiment (see Table 1), which could translate to restoration benefits when planting *Nothofagus* into established *Leptospermum* stands. However, such advantages would need to be tested empirically, ideally by coupling field trials with root tip and soil sampling of ectomycorrhizal communities to determine direct links among the fungal community, host–symbiont preferences, and the competitive success of host plants.

Survival of *Leptospermum* seedlings when subjected to drought stress was also substantially higher when grown with “home” compared to “away” soil inoculum. The drought stress was not severe enough to detect a similar response in *Nothofagus*, but interestingly, survival was highest in the dual‐inoculation treatment for both *Leptospermum* and *Nothofagus*. This could indicate that rather than a specific home soil advantage, the best protection comes from having a wider range of symbiont options. Kipfer et al. (2012) showed that drought tolerance of *Pinus sylvestris* was driven by the presence of particular ectomycorrhizal fungal species rather than overall richness, so it is possible that the wider fungal diversity available in the dual‐inoculation treatment increased the chances of specific fungal species that improve drought tolerance being available. Further experimental research is required to determine which fungal species lead to the greatest tolerance, and where these species naturally occur.

Even if the “home” soil advantage means that fungal communities of other host types are less effective, it is possible that they could provide enough benefits for seedlings to establish initially, after which more preferred communities could accumulate over time via spore dispersal (Dickie et al., 2012; van Galen et al., 2022). We found a positive relationship between colonization and biomass and a negative relationship with richness in *Nothofagus*, which could indicate that ectomycorrhizal colonization, rather than diversity, is the driver of success in *Nothofagus* seedlings during establishment. However, studies examining the fungal richness–plant biomass relationship with other ectomycorrhizal host genera have shown contrasting patterns (Baxter & Dighton, 2001; Sim & Eom, 2006). Long‐term studies tracking changes in fungal community composition to determine the rate or extent to which communities might transition toward those present in mature forests are lacking, and so it is unclear whether *Nothofagus* seedlings establishing in *Leptospermum* shrublands in the field might experience symbiont‐related limitations at some point. Additionally, it is likely that other soil properties altered by *Leptospermum* species such as soil nutrients or bacterial communities could have strong effects on *Nothofagus* seedlings in the field, similar to those observed in *Betula* forests by Liang et al. (2022). We aimed to only manipulate ectomycorrhizal communities in our study (with the underlying soil mix kept constant between treatments) in order to examine the direct effects of fungal communities on plant performance. However, further research should be undertaken to determine how trophic facilitation might be affected by other soil physical and chemical properties.

We found no evidence that when seedlings were inoculated with “away” soil fungi, the presence of the home soil host (i.e., in mixed seedling combinations) improved colonization or changed the community composition of active fungi. Studies of the highly host‐specific ectomycorrhizal fungal genus *Suillus* have shown that some fungal species fail to colonize secondary hosts unless the primary host is also present (Pérez‐Pazos et al., 2021). Although many ectomycorrhizal fungal species do show preferences for certain *Nothofagus* host species (van Galen, Orlovich, Lord, Nilsen, et al., 2023), our results indicate that generalist species are also present in this system that are able to colonize both *Nothofagus* and *Leptospermum*. However, further tests in the absence of the environmental contaminants that affected our experiment would be required to be certain of how the co‐occurrence of host species might influence colonization under field conditions.

That the biomass of control seedlings was sometimes higher than biomass from other treatments indicates that the heat sterilization of soils provided some advantages. Heat sterilization can alter the nutrient properties of soil (Dietrich et al., 2020), however, our methods were designed to avoid this. It is possible that the heat treatment also killed soil pathogens that inhibited growth in the other treatments, leading to greater biomass in the control (Beckstead & Parker, 2003). Alternatively, if a lower number of fungal taxa were available for ectomycorrhizal formation in the control treatment, reduced competition between fungal species may have resulted in ectomycorrhizal associations forming more quickly and efficiently, allowing plants to allocate more resources to growth as shown in other mycorrhizal systems (Pearson et al., 1993). Further experimental studies would be required to untangle these effects.

In conclusion, our results add to the evidence that trophic facilitation has the potential to aid tree establishment, and should therefore be considered when designing restoration practices (Fraser et al., 2015). Despite ambiguity associated with contamination and the fact that *Nothofagus* and *Leptospermum* soil contain different ectomycorrhizal communities, root colonization, richness of active fungi, and plant biomass were comparable regardless of inoculum origin in almost all situations. Therefore, utilizing early‐successional dual‐mycorrhizal species like *Leptospermum* to enrich soil fungal communities prior to establishing late‐successional ectomycorrhizal species like *Nothofagus* should be investigated further as a potential tool for improving restoration. Methods to promote trophic facilitation in restoration could involve actively establishing early‐successional dual‐mycorrhizal shrubs prior to planting ectomycorrhizal‐dependent canopy tree species of interest, sowing seed mixes containing both early‐ and late‐successional species (e.g., Ledgard & Davis, 2004), or by targeting areas for restoration that already contain other ectomycorrhizal hosts (e.g., Davis et al., 2013). Given the diverse characteristics and challenges specific to different systems, detailed field trials are required to determine the most effective course of action.

## AUTHOR CONTRIBUTIONS

**Merissa Strawsine:** Conceptualization (equal); data curation (lead); formal analysis (lead); investigation (equal); methodology (equal); project administration (equal); writing – original draft (equal); writing – review and editing (equal). **Laura G. van Galen:** Conceptualization (equal); data curation (equal); formal analysis (equal); funding acquisition (equal); investigation (equal); methodology (equal); project administration (equal); supervision (equal); visualization (equal); writing – original draft (equal); writing – review and editing (equal). **Janice M. Lord:** Conceptualization (equal); funding acquisition (lead); investigation (equal); methodology (equal); project administration (equal); supervision (equal); writing – original draft (supporting); writing – review and editing (equal). **Matthew J. Larcombe:** Conceptualization (equal); data curation (supporting); formal analysis (supporting); funding acquisition (equal); investigation (equal); methodology (equal); project administration (equal); resources (equal); supervision (equal); writing – original draft (equal); writing – review and editing (equal).

## CONFLICT OF INTEREST STATEMENT

The authors declare no conflicts of interest.

## Supporting information


Data S1.

## Data Availability

Data are provided as Data S1.

## APPENDIX B Quantification of ectomycorrhizal colonization

### B.1

Ectomycorrhizal associations were clearly identified under the dissecting microscope in *Nothofagus* roots due to the presence of the darkly stained mantle sheath and external mycelium. Non‐colonized *Nothofagus* roots were often orange brown in color and unstained by Trypan Blue. *Leptospermum* roots were more difficult to distinguish, so in order to ensure accurate ectomycorrhizal identification, cross sections of root tips with “certain” (dark staining) and “possible” (light staining) ectomycorrhizae were prepared on microscope slides by the University of Otago Department of Histology. Root samples were cut to a thickness of 5 μm using a microtome and stained with hematoxylin and eosin. These cross sections showed the presence of a mantle sheath and Hartig Net in darkly stained roots (indicative of mycorrhizae; Figure B1a,d), but no mycorrhizal structures in lightly stained roots (Figure B1b,c,e,f). Therefore, lightly stained *Leptospermum* roots were considered non‐mycorrhizal.
